# Remarks on Mr. Owen's Letter, Relating to the History of the Venereal Disease

**Published:** 1799-11

**Authors:** William Blair

**Affiliations:** Great Russel Street, Bloomsbury


### *Remarks on Mr.* Owen's *Letter, relating to the Hiſtory of the Venereal Diſeaſe, communicated by Mr.* Blair, *Surgeon of the* Lock Hoſpital *and the* Finſbury Diſpenſary, *&c. &c.*

---

### *To the Editors of the Medical and Phyſical Journal.*

Gentlemen,

In the laſt number of your Journal is a tranſlation of an old Welch manuſcript, ſuppoſed by Mr. Owen and yourſelves "to relate to the "hiſtory of the venereal diſeaſe." It contains a preſcription "for the "great puſtulous eruption and its degrees," as the manuſcript informs us; and "this remedy was ſent by Mr. Ry. Tiler, a French phyſician, "when it was the year of our Lord one thouſand and five hundred, "ſave ſix years," *(i. e.* 1494) "to king Henry VII. the firſt perſon in "this kingdom who was afflicted with that diſeaſe." Admitting, Gentlemen, that yourſelves and your correſpondent do not miſtake the meaning of the original writer, I think the fragment in queſtion muſt be conſidered as a complete refutation of all that has been hitherto advanced concerning the origin and progreſs of the lues venerea. But to me me it ſeems probable, that this fragment has no relation whatever to that diſeaſe; and my opinion is founded on the following conſiderations:

I. The phraſe *venereal diſeaſe* does not occur in the Welch manuſcript, nor is there any analogous expreſſion which can be conſtrued into a reference to that diſorder.

II. The "great puſtulous eruption" with which King Henry VII. was afflicted in 1494, is here deſcribed in ſuch a manner as to prove its diſſimilarity from the lues venerea.

III. Collateral evidence is entirely wanting of this diſeaſe having been introduced into England before the year 1497, ſo that the Welch fragment runs counter to all other hiſtorical evidence.

IV. Upon the ſuppoſition that Henry VII. had actually been infected with the ſyphilis, he was in too elevated a ſtation to eſcape notice among Engliſh hiſtorians. Francis the Firſt, Cæſar Borgia, Cardinal Wolſey, and other perſons of rank, were in this predicament, and their reſpective caſes have been recorded in hiſtory.

V. No preſumption can be taken up in ſupport of Mr. Owen's opinion, merely from the uſe of a mercurial ointment, ſince the ſame remedy was employed in that age againſt leprous affections of the ſkin.

VI. There is no exiſting proof in the annals of medicine, that mercury was uſed externally in ſyphilis ſo early as the year 1494.

On all theſe accounts, therefore, I think it may be fairly concluded, that the Welch manuſcript has been miſunderſtood, and does not relate to the venereal diſeaſe.

May I be permitted, Gentlemen, to avail myſelf of the preſent opportunity of mentioning, for the information of medical men in general, that my *ſecond* collection of caſes to illuſtrate the anti-venereal effects of *nitrous acid*, is not quite completed; and that I ſhall be happy to receive any other caſes which may tend to elucidate this practical enquiry. By inſerting this notice in your next number, you will not only oblige a conſtant reader, but, I truſt, will be ſerving a public cauſe.

*Oct.* 10, 1799, WILLIAM BLAIR.

*Great Ruſſel Street, Bloomſbury.*

